# Guidelines or Other Guidance Documents for Rare Diseases in the Netherlands: When and Where to Invest (Effort, Time and Money)?

**DOI:** 10.1002/gin2.70070

**Published:** 2026-05-12

**Authors:** Iméze J. Hieltjes, Charlotte M. W. Gaasterland, Marieke Hermsen, Anne Marie van Wermeskerken, Ilse van Herk, Johanna H. van der Lee

**Affiliations:** ^1^ Knowledge Institute of the Dutch Association of Medical Specialists Utrecht The Netherlands; ^2^ Amsterdam UMC University of Amsterdam, Emma Children's Hospital Amsterdam The Netherlands; ^3^ Department of Epidemiology Leiden University Medical Centre Leiden The Netherlands; ^4^ Dutch Association of Medical Specialists Utrecht The Netherlands; ^5^ Flevoziekenhuis Almere The Netherlands; ^6^ Dutch Association of Paediatrics Utrecht The Netherlands

## Abstract

**Introduction:**

Developing Clinical Practice Guidelines (CPGs) is resource‐intensive, making it essential to prioritise those CPG projects that are most needed. One of the rules pertains to prevalence, which excludes virtually all guideline development for rare diseases. Still, guidance is needed for their management. We discuss considerations pertaining to the decision to develop a CPG or another type of guidance document for a rare disease.

**Methods:**

Using a consensus‐based approach, an expert group developed a decision support flowchart to decide about the development of a clinical practice guideline or an alternative type of guidance document for rare diseases. An expert group of three guideline methodologists, a paediatrician, and two quality of care policy officers drafted an initial flowchart. The flowchart was reviewed and refined through three rounds of steering committee discussions in different settings, after which final adaptations were made.

**Results:**

The flowchart serves as a support tool to consider whether a CPG is the most suitable type of guidance document for addressing clinical challenges in a particular rare disease. Several alternatives may be more appropriate than developing a CPG, such as a local protocol, a care pathway, a didactic paper, or adolopment or adaptation of an international CPG illustrated by three conditions.

**Discussion:**

The proposed flowchart provides a framework to consider all relevant aspects around the choice for development of a guidance document or CPG. Although it results from the Dutch national situation and the rare disease field, it may also be applied in other countries and for more prevalent diseases. We encourage external validation and, if necessary, refinement of the flowchart. It supports strategic decision‐making, ensuring that the efforts and resources needed for the development of CPGs or other guidance documents are focused on areas where they will have the greatest impact on medical specialist care.

## Summary

- Rare diseases.
- Decision support tool.
- Guideline development.
- Consensus methods.
- Resource allocation.

## 1 Introduction

In Europe, a disease is classified as rare when it affects no more than 1 in 2000 individuals [1]. While each rare disease affects a limited number of people, the collective impact of over 6000 identified rare diseases is substantial, with an estimated 30 million people in Europe living with a rare disease [2]. To improve care for these patients, the European Commission issued a recommendation in 2009 for the establishment and implementation of national plans and strategies to ensure access to high‐quality care [1]. Despite these efforts, many rare diseases still lack Clinical Practice Guidelines (CPGs) or other guidance documents that support clinical decision‐making and enhance the quality of care.

**Figure 1:**
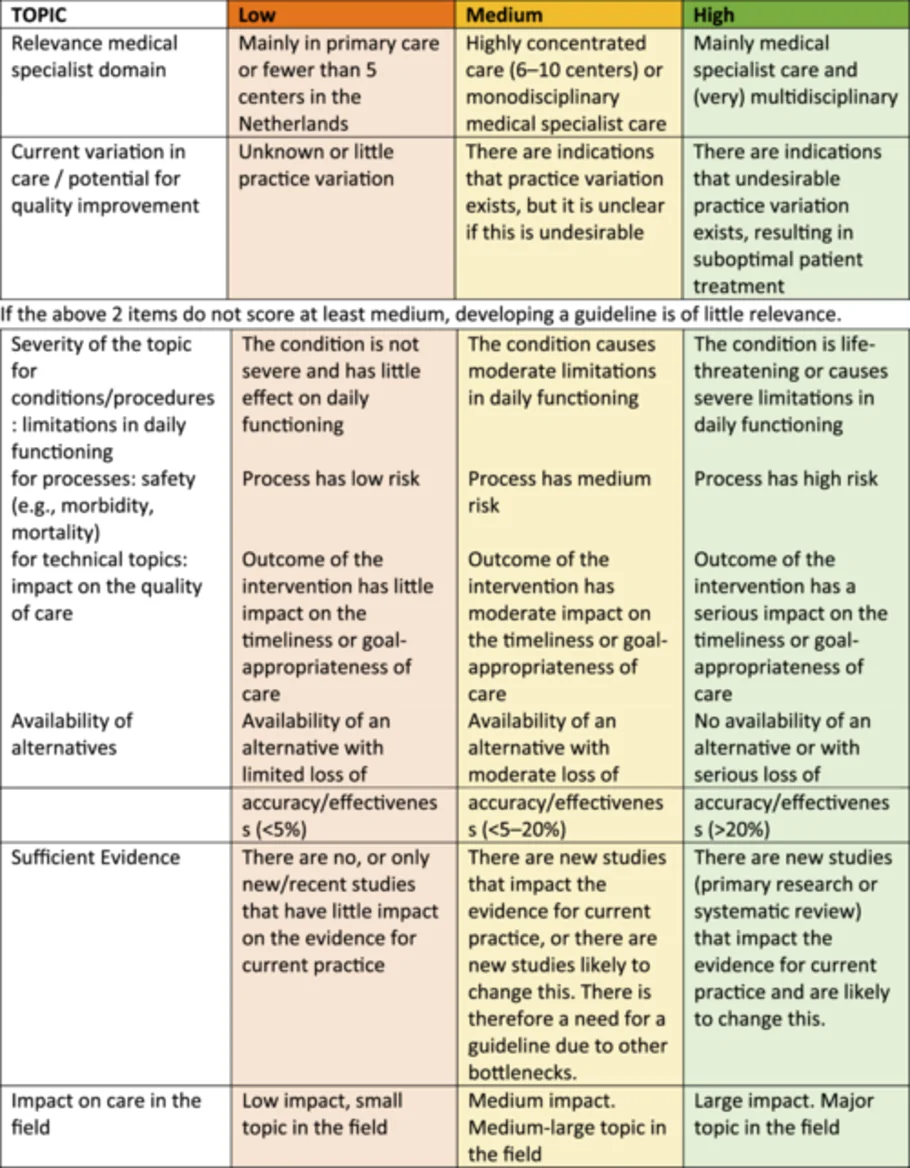
Relevance criteria scheme.

In the Netherlands, the development of national guidelines is funded by the Dutch Medical Specialists Quality Fund (SKMS) and is subject to criteria as determined by the Dutch Association of Medical Specialists (Figure 1) [3]. These criteria include the relevance of the topic in the medical specialist domain, the presence of variation in current practice and potential for quality improvement, the severity and burden of the condition, the availability of sufficient (new) scientific evidence or insights, and the anticipated impact on medical specialist care. Rare diseases typically do not comply with these criteria for funding of CPG development due to their low prevalence, leading to low relevance in the medical specialist domain, since treatment occurs in fewer than five centres in the Netherlands.

If the above 2 items do not score at least medium, developing a guideline is of little relevance.

CPGs are systematically developed documents that include recommendations intended to optimise patient care. They are informed by a systematic review of the best available evidence and an assessment of the benefits and harms of alternative care options [4, 5]. CPGs are developed to reduce unwarranted variations in healthcare, facilitate the translation of evidence into clinical practice, and contribute to improvements in healthcare quality and patient safety. The active involvement of patient representatives in both the development and implementation of CPGs can enhance trust in the process and promote implementation among all relevant interest‐holders [6].

Because of the considerable investments, there should be a balance between the work done by the guideline panel and the number of times a guideline is used in clinical practice. For ultra‐rare conditions, for instance, there may be an ethical constraint to use scarce resources for the development of a guideline that can only be applied to a very small number of patients. On the other hand, the European Commission has stated that every patient should be entitled to the same quality of care, regardless of the prevalence of their disease [7].

Given that developing CPGs for rare diseases is particularly challenging due to limited patient populations, scarce evidence, and the significant time, cost, and labour involved in guideline development [8, 9], it is crucial to prioritise the development of those CPGs that will have the greatest impact on patient care. Due to the limitations of available resources, there is a need to consider alternative types of trustworthy guidance documents that can offer practical guidance to clinicians and patients, but can be developed more parsimoniously.

We use the term guidance document as an umbrella term for other forms of clinical guidance, including care pathways, consensus documents, and local protocols. However, there is no consensus on the terminology for these documents. Moreover, it is often unclear which type of document is most appropriate in a given situation [10]. Depending on the most pressing issues in clinical practice, various instruments may be more appropriate than guidelines. In the context of rare diseases, where formal CPG development is often not feasible, guidance documents may serve as practical alternatives, provided they meet a basic set of methodological standards. Medical specialty societies in the Netherlands regularly receive proposals to develop such documents. This shows the need for a framework that supports decision‐making about the appropriateness and type of document to be developed. Although methodological standards exist for how to develop high‐quality clinical practice guidelines [11, 12], far less guidance is available on when to invest in such development. A structured approach to determine whether guideline development is the most appropriate use of resources is still lacking.

Various types of documents may be needed in different situations. For instance, patients frequently cite diagnostic delay as a key concern; however, such issues may be more effectively addressed through awareness‐raising initiatives than through CPGs [13]. A lack of awareness in medical professionals about particular rare diseases should be alleviated by medical education methods, either in continuing medical education or in (online) didactic papers. Another problem encountered by patients with rare diseases is the absence of effective treatment or limited access to treatment [13]. Challenges like these are influenced by factors beyond the scope of CPGs, including regulatory and economic considerations [14]. Patients sometimes overestimate the influence of CPGs. The absence of a national CPG for a particular disease may be interpreted as a lack of recognition of their particular disease. However, as stated before, the development and updating of CPGs for each known rare disease is impossible.

In the Netherlands, the care for many rare diseases is concentrated in one or two centres of expertise. In these cases, local treatment or research protocols can effectively serve the function of a ‘national’ guideline, provided they are developed in close collaboration with patients and based on a thorough analysis of the scientific literature [3]. Initiatives such as the European Reference Networks (ERNs), aiming to facilitate international collaboration for rare diseases, can enhance the development of international CPGs for rare diseases, but there will always be limitations to the available human and financial resources.

The development of the flowchart took place within a broader national project on guidance development for rare diseases. The overarching aim of this project was to explore which alternative approaches to traditional clinical practice guidelines are most appropriate in the context of rare diseases, and how these approaches can support maintenance and improvement of quality of care while responsibly addressing the introduction of often costly innovations. As such, the project focused on safeguarding and strengthening the quality cycle for patients with rare conditions.

During the course of this project, recurring discussions arose regarding terminology and proportionality, particularly concerning the distinction between clinical practice guidelines and other forms of guidance documents. These discussions highlighted the need for a structured approach to decision‐making about when investment in full guideline development is justified and when alternative forms of guidance are more appropriate.

In response to this need, we developed a flowchart outlining key considerations for deciding whether to develop a CPG or another type of guidance document for a rare disease.

## 2 Methods

### 2.1 Study Design and Objective

The flowchart was developed using an iterative expert consensus approach, combining methodological expertise in guideline development with input from clinical specialists, patient representatives, and policy stakeholders.

### 2.2 Development of the Initial Draft

In 2022, a project group consisting of three guideline methodologists, one paediatrician with expertise in guideline development, and two quality of care policy officers from the Dutch Paediatric Association and the Dutch Association of Medical Specialists drafted an initial version of the flowchart. This draft was informed by the existing relevance criteria for guideline development as defined by the Dutch Association of Medical Specialists, which are used nationally to prioritise topics for funded guideline development.

### 2.3 Steering Committee Review and Consensus Process

The initial draft of the flowchart was reviewed by the steering committee comprising 23 representatives from Dutch medical professional societies and national patient organisations. The committee members represented a broad range of medical specialties and stakeholder perspectives relevant to rare disease care and guideline development.

Organisations represented in the steering committee:

**Table:** 

Patient umbrella organisation for rare and genetic disorders (Netherlands)
Child and hospital foundation
Guidelines Advisory Board of the Dutch Association of Medical Specialists
Dutch Association of Paediatrics
Association of Clinical Genetics Netherlands
Dutch Society for Obstetrics and Gynaecology
Dutch Society of Internal Medicine
Netherlands Society of Neurology
Dutch Society for Neuromuscular diseases
Netherlands Nurses and Nursing Assistants Association
Association of Innovative Medicines
Dutch Ophthalmological Society
Dutch Association of Anaesthesiology
Dutch Association of Physicians for Pulmonary Diseases and Tuberculosis
Dutch Society of Dermatology and Venereology
Netherlands Society for Clinical Chemistry and Laboratory Medicine
Dutch Association of Plastic Surgery
Dutch Society for Rheumatology
Dutch society of Cardiology
Dutch Society for Surgery
Dutch Association of Hospital Pharmacists
Netherlands Society of Rehabilitation Medicine

The consensus process was organised within an ongoing project governance structure and involved multiple groups with distinct roles. An iterative core development process took place within a small project group, which met repeatedly to draft, revise, and refine the flowchart.

The draft flowchart was discussed once in an online meeting with the project steering committee, where feedback was provided on the terms and the order of the steps. In addition, the draft was shared once with 20 colleagues involved in guideline methodology within the Knowledge Institute of the Dutch Association of Medical Specialists for informal peer review. Here, the order of the steps and clarifications of terminology were also discussed. Finally, the flowchart was discussed during a single plenary meeting with representatives of all 32 medical professional societies affiliated with the Dutch Association of Medical Specialists, allowing for broader stakeholder input.

No formal voting procedures were used. Feedback was provided verbally during meetings and changes were made by the project group when considered relevant. Revisions across meetings primarily concerned clarification of decision steps, distinctions between clinical practice guidelines and other types of guidance documents, and the practical sequencing of the flowchart.

Finally, the flowchart was applied to three rare disease cases (spinal muscular atrophy type II/III, cutaneous lymphomas, and Barth syndrome) to assess its practical usability and feasibility in supporting decision‐making about the development of different types of guidance documents.

## 3 Results

The first version of the flowchart was discussed with the project steering committee (Appendix 1). At this and subsequent stages, proposed changes were reviewed within the project group and incorporated when considered relevant and of added value. Revisions primarily concerned clarification of decision points, differentiation between types of guidance documents, and the practical sequence of steps within the flowchart.

**Figure 2:**
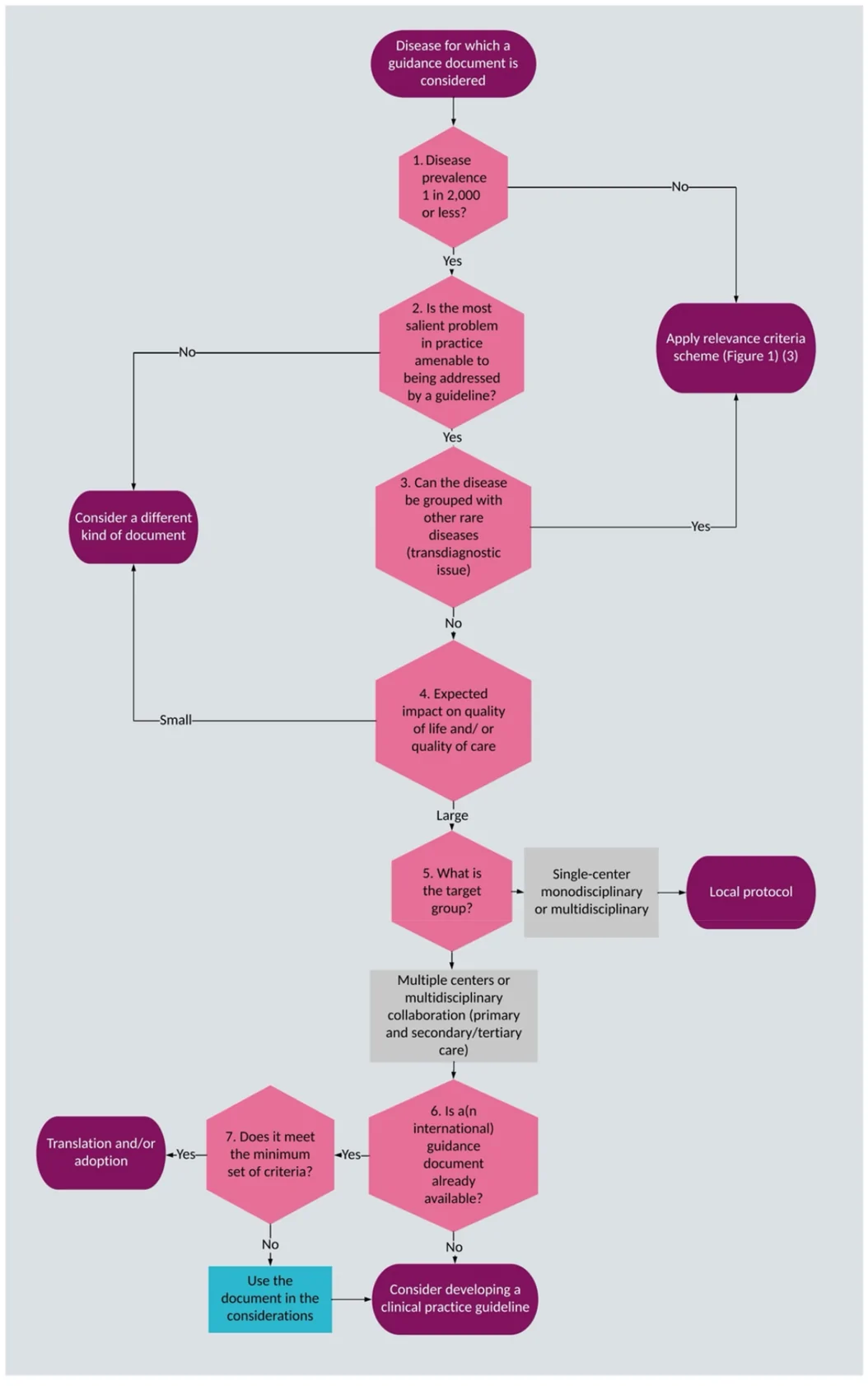
Flowchart for consideration whether development of a CPG or other type of guidance document is warranted.

After several revisions based on input from all groups involved, we propose the following flowchart (Figure 2).

Flowchart decisions:

1. A rare disease is defined as a condition that affects no more than 1 in 2000 individuals. This threshold is used as the first step in the flowchart to exclude non‐rare diseases.
2. If it is not clear what is the most salient problem in practice, an inventory can be made among all interest‐holder groups. This can be done by email questionnaires or by organising an invitational conference, in which all interest‐holder groups are invited to list the problems they encounter in practice. The initiators of the project should then assess whether the problems on this list are relevant and whether a CPG can solve one or more problems on the list. CPGs can provide solutions to challenges experienced by healthcare professionals and patients, such as inconsistencies in treatment approaches between hospitals (unwarranted practice variation) or uncertainty regarding the best treatment options. However, some challenges cannot be effectively addressed by a guideline. Examples include:

- Fragmented care: When a specific procedure is performed in many centres, this may result in insufficient development and conservation of expertise. If this is the case, professionals need to establish agreements on the centralisation of care and the referral to expert centres.
- Diagnostic delay: When clinicians are not familiar with a condition, this may result in diagnostic delay. This issue may be better addressed by continuing medical education or by other means to raise awareness rather than a guideline.

3. If a specific symptom or issue is relevant to multiple rare diseases, it may be more efficient to develop a guideline for an overarching or transdiagnostic problem rather than for a single disease. This approach is particularly useful when multiple rare diseases share similar challenges or when common symptoms require structured guidance, e.g. sleep disturbances, the need for mechanical ventilation, or the transition from paediatric to adult care.
4. Evaluating the potential impact of a guideline on the quality of life and quality of care is crucial in determining whether its development is justified. If the expected impact is minimal, investing in the development of a guideline may not be advisable given the considerable resources required. Of course, this is an arbitrary decision, which depends on the perspective of the persons involved and on the availability of resources.
5. If a guidance document is intended for a condition managed by one or more disciplines within a single centre, a local protocol may be more suitable and efficient than a national guideline. However, if the document is intended for multiple centres or involves both primary and secondary/tertiary care, a national (or even international) guideline may be more appropriate. It is important to assess whether the issue can be solved with a local protocol or whether broader interest‐holder involvement and a more extensive methodology, such as that used for guideline development, is required.
6. If all previous steps in the flowchart indicate the need for a guideline and no existing national or international guideline is available for the rare disease in question, developing a new guideline may be warranted, provided that sufficient funding can be secured.
7. If a guidance document does exist, particularly at the international level, it is essential to assess whether it meets a minimum set of quality criteria. This minimum set of criteria can help users to assess the trustworthiness of guidance documents for rare diseases. If the existing guideline does not meet these criteria, its content may still be useful in informing the development of a new guideline. However, if the existing document meets the quality standards and does not require translation or adaptation, implementing the available guideline is recommended rather than developing a new one [15].

Table 1 shows how these decisions were made in the examples of SMA, cutaneous lymphoma and Barth syndrome.

**Table 1:** Application of the flowchart for SMA, Cutaneous Lymphomas and Barth Syndrome.

Step	SMA II/III	Cutaneous lymphoma	Barth syndrome
1. Rare?	+	+	+
2. Most salient problem amendable by CPG?	+ (Physical training)	+ (Allogeneic stem cell transplantation)	‐
3. Grouping possible?	‐	‐	‐
4. Impact	Large	Large	‐
5. Target group	Primary, secondary and tertiary care	Secondary and tertiary care	Single centre
6. International guidance document available?	‐	+*	‐
7. Does it meet the minimum criteria?			
Conclusion	CPG	Use of international CPG	Different type of document

* Only later was found out that an international CPG had been submitted for publication.

Spinal Muscular Atrophy is a rare genetic neuromuscular disease (birth prevalence < 1:10,000), ranging in severity from type I (first symptoms before 6 months of age, affected children do not develop the ability to sit) to type IV (first symptoms at adult age, normal life expectancy). For SMA type II/III there was no national guideline or other guidance document, but there was a clear clinical challenge related to primary, secondary, and tertiary care. With the introduction of effective disease‐modifying treatments, the general prognosis for patients with SMA has improved; however, considerable uncertainty remains about the positive or harmful effects of physical training. Physical training is generally supervised by physiatrists and physical therapists in the patient's home environment, rather than in the only expert centre in the country. Consequently, a CPG was developed and published on the national guidelines database with recommendations for these disciplines [16]. For cutaneous lymphomas, a group of rare malignant skin disorders mainly occurring in adults (prevalence < 1:100,000), there is also only one expert centre in the Netherlands. However, most patients are managed by dermatologists in secondary care. Therefore, this disease group was chosen for the pilot project. At that time, the project group was not aware that an international guideline had just been completed, albeit not published yet [17]. Therefore, no guidance document was developed.

Barth syndrome is an X‐linked genetic metabolic disorder (birth prevalence < : 100,000). Medical care for this disease is highly concentrated in a single expert centre in the Netherlands. The experts from this centre used the flowchart to decide on the type of guidance document they would develop. They concluded that a care pathway, defined as a structured and coordinated process within the healthcare system, designed to ensure optimal and consistent patient care [18], was considered proportional to the available resources and scope of clinical need.

## 4 Discussion

Here we present a structured framework to support targeted decision‐making about whether to develop a CPG or another type of guidance document for rare diseases. Given that most rare diseases are not eligible for funded guideline development in the Netherlands, and probably in many other countries, this tool helps in deciding on the most appropriate and feasible approach. By clarifying when a CPG is warranted and when alternative, less resource‐intensive guidance documents may suffice, the flowchart supports informed choices about how best to address specific clinical challenges.

While not required for every disease, CPGs may have the most added value for diseases where significant practice variability exists and for which a valid evidence base can guide recommendations [19, 20]. A guideline is most valuable when it addresses a problem area in medical care, such as a lack of consensus about clinical management among medical specialists, controversy regarding best practices, or unwarranted practice variation. In the Netherlands, the identification of such problem areas provides the basis for guideline development. This focus ensures that recommendations are targeted and relevant. Integrating this principle into early decision‐making helps ensure that guideline development efforts are both efficient and aligned with genuine clinical needs. By highlighting when guideline development is not the most effective option, the flowchart supports a more targeted allocation of resources and prevents unnecessary spending of human and financial resources.

The three rare disease applications were intended to explore feasibility and decision logic rather than to evaluate outcomes or impact. For spinal muscular atrophy type II/III, the flowchart supported the development of a national clinical practice guideline, addressing uncertainties in physical training and ensuring consistent recommendations across primary, secondary, and tertiary care. In contrast, for cutaneous lymphomas, the flowchart indicated that the adoption or adaptation of an existing high‐quality international guideline was more efficient than initiating a new national guideline. Finally, for Barth syndrome, where care is concentrated in a single expert centre, and patient numbers are very small, the flowchart supported the decision to develop a local protocol rather than a guideline. These examples support the relevance and usability of the flowchart in diverse rare disease contexts.

This study has several limitations. First, the flowchart was developed as an emergent product of a broader project aimed at advising the Dutch Association of Medical Specialists on the role of different types of guidance documents in rare diseases, rather than as a stand‐alone study with a prespecified protocol. As a result, no formal development or validation framework was defined a priori. Moreover, the development of this flowchart was primarily aimed at the Dutch healthcare setting, which may limit its generalisability.

Second, although the flowchart was iteratively discussed and refined in multiple meetings with representatives from all Dutch medical specialist societies and with guideline development professionals from the Knowledge Institute, it has not been formally evaluated or validated. The flowchart should therefore be regarded as a tool intended to structure and increase transparency in decisions about whether to invest in the development of clinical practice guidelines or alternative forms of guidance, rather than as a final decision tool. Even though the flowchart was developed for the Dutch setting, it may still be relevant in other (European) countries that face similar financial and human constraints. European Reference Networks (ERNs) aim to harmonise care internationally, and this framework could support decisions at that level too. While designed for rare diseases, the underlying considerations are not exclusive to this group. Common diseases that face similar issues, such as lack of evidence or fragmented care, could also benefit from a structured approach to selecting the appropriate type of guidance document to be developed. One of the most critical aspects when considering a guidance document is identifying the specific clinical problem area, step 2 in the flowchart.

Further development of the flowchart might include its application in other countries, and also in the non‐rare disease context, to assess whether it supports decisions as consistently as in the example diseases presented in this paper. In addition, international consensus on definitions and on the classification of the various types of guidance documents would enhance clarity and ensure consistent use across projects.

Although considerable progress has been made in advancing methods for producing high‐quality guidelines, significantly less attention has been given to establishing criteria for selecting appropriate topics and determining the optimal allocation of limited resources. The question of what type of guidance to develop, for which topics, and under what conditions remained largely unaddressed thus far. With this flowchart, we aimed to take a first step toward filling this gap by offering a structured tool to support these decisions. We consider this a missing piece in the current guideline methodology, and we invite others to further refine and build upon this approach to improve strategic prioritisation in guideline development.

## Author Contributions

**Iméze J. Hieltjes:** writing – original draft preparation, conceptualisation, investigation. **Charlotte M. W. Gaasterland:** writing – review and editing, conceptualisation, investigation. **Marieke Hermsen:** writing – review and editing, conceptualisation. **Anne Marie van Wermeskerken:** writing – review and editing, conceptualisation. **Ilse van Herk:** writing – review and editing, conceptualisation. **Johanna H. van der Lee:** writing – review and editing, conceptualisation, investigation.

## Funding

The authors have nothing to report.

## Conflicts of Interest

The authors declare no conflicts of interest.

## Supporting information

Supporting File

## Data Availability

The authors have nothing to report.
