# Supplementary material for: Guidelines or Other Guidance Documents for Rare Diseases in the Netherlands: When and Where to Invest (Effort, Time and Money)?
Source: Clin Public Health Guidel. 2026 May 12;3(3):e70070. doi: 10.1002/gin2.70070 (PMC13563323; doi:10.1002/gin2.70070)
Supplement: Supplementary file 1 [file GIN2-3-e70070-s001.pdf]

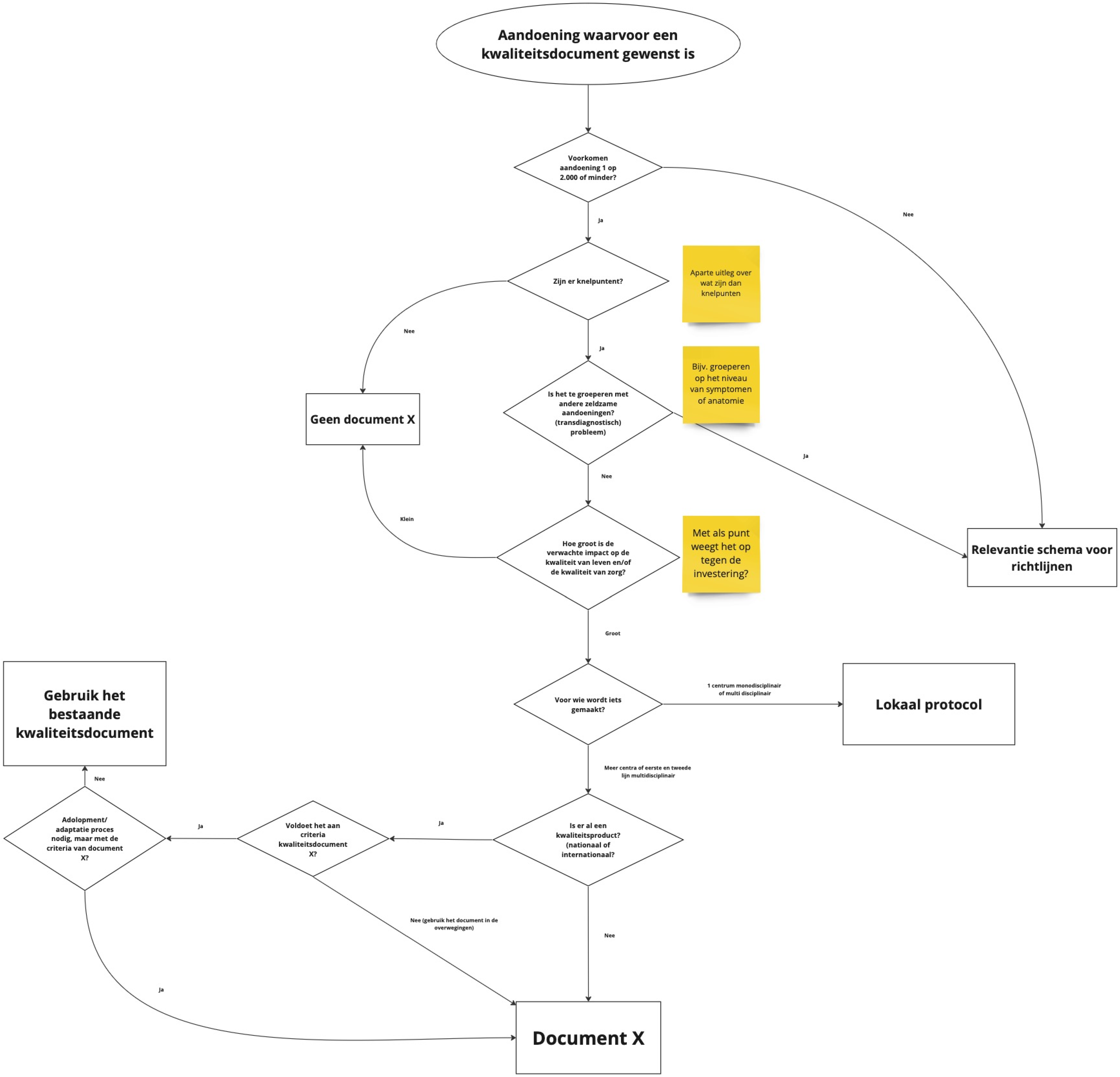
Aandoening waarvoor een kwaliteitsdocument gewenst is
Voorkomen aandoening 1 op 2.000 of minder?
Nee
Ja
Zijn er knelpuntent?
Aparte uitleg over wat zijn dan knelpunten
Nee
Ja
Is het te groeperen met andere zeldzame aandoeningen? (transdiagnostisch) probleem)
Bijv. groeperen op het niveau van symptomen of anatomie
Geen document X
Ja
Nee
Klein
Hoe groot is de verwachte impact op de kwaliteit van leven en/of de kwaliteit van zorg?
Met als punt weegt het op tegen de investering?
Relevantie schema voor richtlijnen
Groot
Gebruik het bestaande kwaliteitsdocument
Voor wie wordt iets gemaakt?
1 centrum monodisciplinair of multi disciplinair
Lokaal protocol
Meer centra of eerste en tweede lijn multidisciplinair
Nee
Adolopment/ adaptatie proces nodig, maar met de criteria van document X?
Ja
Voldoet het aan criteria kwaliteitsdocument X?
Ja
Is er al een kwaliteitsproduct? (nationaal of internationaal?
Nee (gebruik het document in de overwegingen)
Nee
Ja
Document X
